# Efficient non-contrast enhanced 3D Cartesian cardiovascular magnetic resonance angiography of the thoracic aorta in 3 min

**DOI:** 10.1186/s12968-021-00839-9

**Published:** 2022-01-10

**Authors:** Anastasia Fotaki, Camila Munoz, Yaso Emanuel, Alina Hua, Filippo Bosio, Karl P. Kunze, Radhouene Neji, Pier Giorgio Masci, René M. Botnar, Claudia Prieto

**Affiliations:** 1 Department of Biomedical Engineering, School of Biomedical Engineering and Imaging Sciences, King’s College London, St Thomas’ Hospital, 3rd Floor-Lambeth Wing, Westminster Bridge Road, London, SE1 7EH UK; 2 Department of Cardiology, NHS Foundation Trust, Guy’s and St Thomas, London, UK; 3 MR Research Collaborations, Siemens Healthcare Limited, Frimley, UK; 4 Escuela de Ingeniería, Pontificia Universidad Católica de Chile, Santiago, Chile

**Keywords:** Undersampled Cartesian MRA, iNAV, Thoracic aortic disease, Non-rigid motion correction

## Abstract

**Background:**

The application of cardiovascular magnetic resonance angiography (CMRA) for the assessment of thoracic aortic disease is often associated with prolonged and unpredictable acquisition times and residual motion artefacts. To overcome these limitations, we have integrated undersampled acquisition with image-based navigators and inline non-rigid motion correction to enable a free-breathing, contrast-free Cartesian CMRA framework for the visualization of the thoracic aorta in a short and predictable scan of 3 min.

**Methods:**

35 patients with thoracic aortic disease (36 ± 13y, 14 female) were prospectively enrolled in this single-center study. The proposed 3D T2-prepared balanced steady state free precession (bSSFP) sequence with image-based navigator (iNAV) was compared to the clinical 3D T2-prepared bSSFP with diaphragmatic-navigator gating (dNAV), in terms of image acquisition time. Three cardiologists blinded to iNAV vs. dNAV acquisition, recorded image quality scores across four aortic segments and their overall diagnostic confidence. Contrast ratio (CR) and relative standard deviation (RSD) of signal intensity (SI) in the corresponding segments were estimated. Co-axial aortic dimensions in six landmarks were measured by two readers to evaluate the agreement between the two methods, along with inter-observer and intra-observer agreement. Kolmogorov–Smirnov test, Mann–Whitney U (MWU), Bland–Altman analysis (BAA), intraclass correlation coefficient (ICC) were used for statistical analysis.

**Results:**

The scan time for the iNAV-based approach was significantly shorter (3.1 ± 0.5 min vs. 12.0 ± 3.0 min for dNAV, P = 0.005). Reconstruction was performed inline in 3.0 ± 0.3 min. Diagnostic confidence was similar for the proposed iNAV versus dNAV for all three reviewers (Reviewer 1: 3.9 ± 0.3 vs. 3.8 ± 0.4, P = 0.7; Reviewer 2: 4.0 ± 0.2 vs. 3.9 ± 0.3, P = 0.4; Reviewer 3: 3.8 ± 0.4 vs. 3.7 ± 0.6, P = 0.3). The proposed method yielded higher image quality scores in terms of artefacts from respiratory motion, and non-diagnostic images due to signal inhomogeneity were observed less frequently. While the dNAV approach outperformed the iNAV method in the CR assessment, the iNAV sequence showed improved signal homogeneity along the entire thoracic aorta [RSD SI 5.1 (4.4, 6.5) vs. 6.5 (4.6, 8.6), P = 0.002]. BAA showed a mean difference of < 0.05 cm across the 6 landmarks between the two datasets. ICC showed excellent inter- and intra-observer reproducibility.

**Conclusions:**

Thoracic aortic iNAV-based CMRA with fast acquisition (~ 3 min) and inline reconstruction (3 min) is proposed, resulting in high diagnostic confidence and reproducible aortic measurements.

**Supplementary Information:**

The online version contains supplementary material available at 10.1186/s12968-021-00839-9.

## Background

Cardiovascular magnetic resonance angiography (CMRA) is an established diagnostic tool for serial monitoring of thoracic aortic disease, which encompasses a broad range of degenerative, structural (i.e. aortic coarctation, aortic valvar stenosis, bicuspid aortic valve), acquired (i.e. hypertension, chronic obstructive pulmonary disease, smoking and inflammatory conditions), genetic-based (i.e. Marfan syndrome, Ehlers-Danlos syndrome, Loeys-Dietz syndrome), and traumatic disease states and presentations [1–3]. There are an estimated 3000–8000 new cases of chronic thoracic aortic aneurysms in the UK each year [4].

Thoracic aortic disease is usually asymptomatic and not easily diagnosed until an acute and life-threatening complication occurs, including dissection and/or rupture [5]. Serial, life-long, robust aortic imaging is crucial to allow for monitoring of the disease progress, pre-procedural planning and post-procedural follow-up [6, 7].

Several cardiovascular magnetic resonance (CMR) techniques have been proposed for the assessment of aortic disease, including contrast-enhanced (CE) CMRA [8, 9] and CE balanced steady-state free precession (bSSFP) sequences [10]. Various impediments associated with the aforementioned techniques, including concerns regarding deposition after repeated administration of gadolinium-based contrast agent and the extremely low risk of nephrogenic systemic fibrosis in patients with kidney disease, have shifted clinicians’ interest towards non-contrast CMRA sequences (NC-CMRA) [1, 11–13]. Recent studies have shown that electrocardiogram (ECG)-triggered, free breathing diaphragmatic-navigator gated CE [14] or NC T2-prepared bSSFP sequences can be applied for aortic disease monitoring [10, 13, 15–18]. These sequences offer inherent high contrast between blood and background tissues, thereby allowing for optimal delineation of the aortic lumen [8, 19] and accurate, reproducible pre-operative monitoring of aortic root diameters [1, 15, 20, 21]. However, several limitations are intrinsic to this approach [22, 23]. Conventional 3D bSSFP CMRA techniques use diaphragmatic respiratory gating (dNAV) to minimize the effects of respiratory motion in the images, which results in unpredictable and excessively long acquisition times [22] and the risk of incomplete or aborted scans because of respiratory drift [23, 24]. Alternatively, a variety of 1D (one-dimensional) self-navigated techniques have been proposed [18, 25–27], which allow the extraction of a foot-head respiratory motion signal directly from the image data that can be used for translational respiratory motion correction during post-processing. However, 1D self-navigation also suffers from certain limitations that are related to the 1D translational motion model used for correction, which may not be accurate when a wide range of respiratory motion is present, such as the case among the different thoracic aortic components [28–30]. During the respiratory cycle, the thoracic aorta experiences a consequent displacement that follows the respiratory excursions of the thoracic wall, with significantly smaller displacement for the descending aorta than that of the ascending segment [29]. 3D-rendered computed tomography angiography (CTA) images and lumen models for patients with aortic pathology have shown that significant multi-directional translation occurred secondary to respiration, shifting the thoracic aorta and arch vessels both posteriorly and superiorly [30].

Translational respiratory motion correction using low-resolution 2D (two-dimensional) or 3D (three-dimensional) image-based navigators (iNAVs) has been applied for coronary MRA [31, 32]. With these approaches, the heart can be spatially isolated from surrounding static tissues and 2D/3D translational respiratory motion can be estimated and corrected in a beat-to-beat fashion. To correct for remaining non-rigid respiratory-induced cardiac motion, and thus to further improve coronary CMRA image quality, techniques that include non-rigid motion correction based on respiratory binning techniques have been proposed [33, 34]. In this approach, the acquired data are assigned to several states of the breathing cycle (or “bins”) and are later corrected to a reference position using non-rigid motion estimated from the binned images. While this approach has been previously demonstrated for high-resolution coronary CMRA from a ~ 10 min scan, its use for aortic imaging has not been explored so far.

The aim of this study was to extend this approach to NC thoracic CMRA in order to achieve highly resolved visualization of the thoracic aorta at 1.6 mm^3^, in a short and predictable scan of ~ 3 min, with a rapid, fully inline non-rigid motion corrected undersampled reconstruction in 3 min for fast clinical testing and adoption. The feasibility of this approach was evaluated in patients with different aortic pathologies, and its performance in terms of image quality and overall acquisition time was compared to clinical NC 3D T2-prepared bSSFP MRA with dNAV gating.

## Methods

### Study design

This was a prospective single center study. The study was approved by the local institutional review board and the UK National Research Ethics Committee.

### Study population

The study population consisted of patients referred for a clinically indicated CMRA between the December 1, 2020 and November 1, 2021. Specific exclusion criteria included: patients with contraindications for CMR (e.g. pacemaker, cochlear implants, cerebral aneurysm clip, implanted electronic device and claustrophobia) and inability to lie flat.

### Data acquisition

Thirty-five patients, with a clinically indicated thoracic aorta CMRA, were scanned under free breathing on a 1.5 T clinical CMR scanner (Magnetom Aera, Siemens Healthineers, Erlangen, Germany). Data were acquired with an 18-channel chest coil and a 32-channel spine coil. Written informed consent was obtained from all subjects according to institutional guidelines.

Two acquisitions were performed for the assessment of the thoracic aorta. First, a standard clinical T2-prepared 3D whole-heart dataset was acquired with the following imaging parameters: field-of-view (FOV) 400 × 227–307 × 140–179 mm, spatial resolution 1.6 × 1.6 × 1.6 mm^3^, T2-prepation duration = 40 ms, GRAPPA parallel imaging 2× undersampled, flip angle = 90◦, TR(repetition time)/TE(echo time) 3.5 ms/1.72 ms, receiver bandwidth 930 Hz/px, sagittal orientation. For the T2-preparation pulse, a typical preparation module (as per our clinical setting) consisting of a train of two equally-spaced composite refocusing pulses with Malcolm-Levitt phase cycling (MLEV2) has been applied. This sequence was combined with a conventional dNAV for respiratory motion compensation, with a gating window of ± 3.5 mm in end-expiration and slice tracking factor of 0.6. After the clinical sequence, data were acquired with the proposed method, which is briefly described below.

**Fig. 1:**
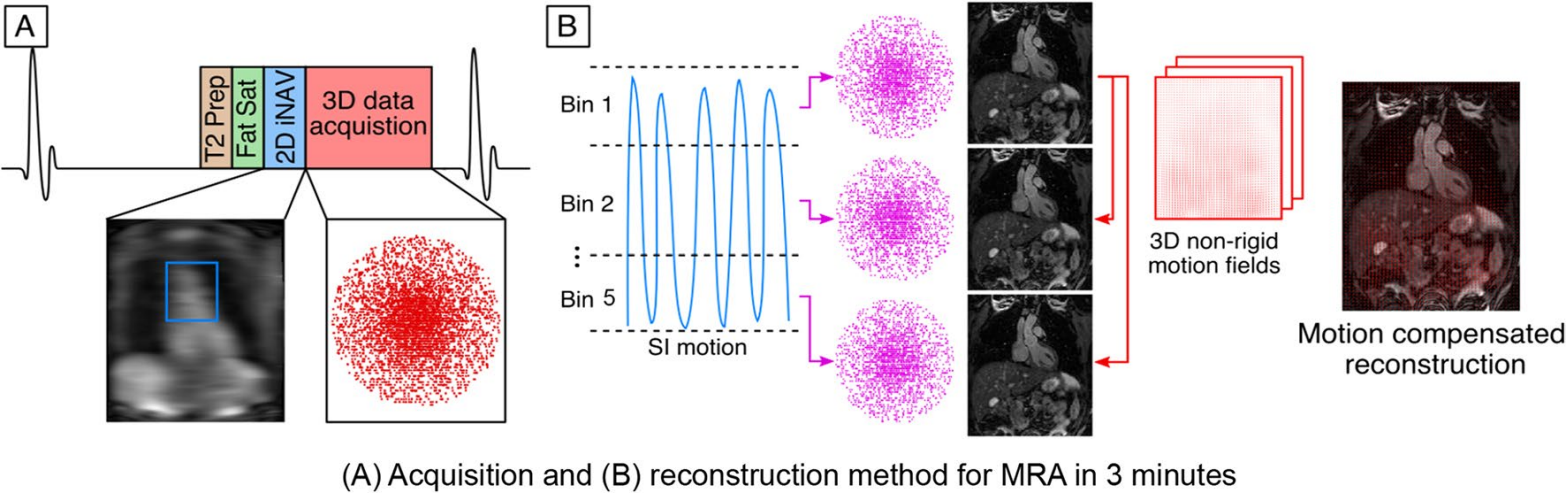
Diagram showing the proposed electrocardiogram (ECG)-triggered free-breathing 3D T2-prepared balanced steady state free precession (bSFFP) sequence with a threefold undersampled variable-density Cartesian trajectory. 2D image-based navigators (iNAV)s are acquired at each cardiac cycle by spatially encoding the start-up echoes preceding the 3D cardiovascular magnetic resonance angiography (CMRA) acquisition to enable 100% scan efficiency and predictable scan time. Fat saturation and adiabatic T2 preparation pulses are used to improve signal homogeneity in the blood pool without using exogenous contrast agents (**A**). The iNAVs. are used to estimate foot-head and right-left (RL) rigid motion by tracking a template around the aortic arch, providing motion estimates in a beat-to-beat basis. Foot-head motion is used to sort the 3D CMRA data in 5 equally populated bins, and 3D CMRA images reconstructed at each respiratory position are used to estimate non-rigid motion between bins (**B**). 2D translational beat-to-beat and 3D non-rigid bin-to-bin motion is then integrated into an in-line motion-compensated iterative SENSE reconstruction to produce the final images

The proposed sequence consists of an ECG-triggered free-breathing 3D T2-prepared bSFFP prototype sequence with a threefold undersampled variable-density Cartesian trajectory (Fig. 1). 2D iNAVs. are acquired at each cardiac cycle by spatially encoding the start-up echoes preceding the 3D CMRA acquisition to enable 100% scan efficiency (no data rejection during the respiratory cycle) and predictable scan time. Fat saturation and four equally-spaced composite refocusing pulses with Malcolm-Levitt phase cycling (MLEV4) [35, 36] are used to improve contrast between blood pool and surrounding tissues, and to improve signal homogeneity in the blood pool without using exogenous contrast agents (Fig. 1A). The iNAVs are used to estimate foot to head (FH) and right-left (RL) rigid motion by tracking a template around the aortic arch, providing motion estimates in a beat-to-beat basis. FH motion is used to sort the 3D CMRA data in five equally populated bins, and 3D CMRA images reconstructed at each respiratory position are used to estimate non-rigid motion between bins (Fig. 1B). 2D translational beat-to-beat and 3D non-rigid bin-to-bin motion is then integrated into an in-line motion-compensated iterative Sensitivity Encoding (iterative SENSE) reconstruction [33] to produce the final images. Imaging parameters for the proposed sequence include, FOV: 400 × 300 × 115–140 mm, spatial resolution 1.6 × 1.6 × 1.6 mm^3^, flip angle = 90°, T2-preparation duration = 40 ms, TE/TR = 1.72/3.44 ms, receiver bandwidth 558 Hz/px, coronal orientation. No heart rate lowering drugs were given to the subjects, and the iNAV acquisition was always performed after the clinical CMRA scan. Cine imaging was acquired in the transverse orientation to determine the subject specific mid‐diastolic resting period (cardiac acquisition window ~ 120–150 ms). For both dNAV- and iNAV-based sequences the acquisition window was manually set, depending on the the subject specific mid‐diastolic resting period. Acquisition times for both aortic CMRA scans were recorded.

### Qualitative image quality analysis

Image quality of the proposed iNAV T2prepared bSSFP and conventional dNAV T2prepared bSSFP datasets were assessed by three cardiologists (YE, AF and AH, 15, 3 and 2 years of experience in CMR, respectively, YE and AF hold Level 3 Certification in CMR). Seventy whole-heart image datasets were randomized and de‐identified for evaluation. The three readers jointly reviewed five anonymized datasets to calibrate their scores together. Following this training session, each reader was blinded to image acquisition type, the other reader, and clinical history for independent evaluation of all the datasets.

Overall diagnostic confidence of the iNAV datasets versus the conventional dNAV images was assessed by the three reviewers. Diagnostic information was related to: (a) exclusion of diseases, (b) diagnosis of suspected abnormalities and (c) diagnosis of unsuspected abnormalities relevant to the patient’s assessment regarding aortopathy. Level of confidence to assess the 3D relationships between structures (score of 1 = poor image quality, poorly defined anatomic details, poor diagnostic confidence; score of 2 = reduced image quality, limitations in anatomic detail, impairment of diagnostic confidence; score of 3 = good image quality, clear anatomic details, no impairment of diagnostic confidence; score of 4 = excellent image quality, distinct anatomic details, full diagnostic confidence) was recorded.

A more differentiated analysis of subjective image quality of CMRA was performed and specific pre-defined anatomical structures were assessed: Aortic Root (ARoot), Mid Ascending Aorta (mAA), Mid aortic arch (mAAr), Mid Descending Aorta (mDA). The image quality assessment was based on a 4-point scoring system and was divided on homogeneity of signal in the blood pool (1 = non diagnostic, 2 = poor, 3 = adequate-good, 4 = excellent) and presence of artefacts due to respiratory motion (1 = severe artefact, 2 = significant artefact, 3 = mild artefact, and 4 = minimal artefact). For each observer, the scores of the two imaging techniques were compared using a Mann–Whitney U-test.

### Quantitative image quality analysis

To quantify the inhomogeneity of the blood pool signal, the relative standard deviation (RSD) of the signal intensity was computed in a circular region-of-interest (ROI) of 0.5 cm^2^ placed ARoot, mAA, mAAr, mDA. The RSD in percent is simply defined as the standard deviation of the voxel intensities in the ROI (σROI) divided by their mean value (µROI): RSD = 100 × σROI/µROI [%] [23]. Additionally, the contrast between blood and myocardium was measured by comparing the signal intensities (SI) in the above-mentioned blood ROIs to those from ROIs at the anterior part of the myocardium of the left-ventricle at a mid-ventricular level. The blood–myocardium contrast ratio (CR) was computed as (µROI, blood − µROI, myocardium)/µROI, myocardium).

Aortic dimensions were measured at six landmarks predefined by literature guidelines, ARoot, sinotubular junction (STJ), mAA, mAAr, mDA, distal descending aorta at the level of the diaphragm (dDAo) [1]. Diameter measurements were performed perpendicular to the longitudinal or flow axis of the aorta to correct for the variable geometry of the aorta, as suggested by current guidelines [1]. The aortic root was cusp-commissure measured. mAAo and mDAo were measured at the level of the right pulmonary artery, the diameter at the mAAr was measured between the left common carotid and the left subclavian artery, the diameter at the distal descending aorta was measured at the level of the diaphragm, in agreement with the literature [1]. Blinded co-axial measurements (maximum diameter) were performed using multiplanar reformats (Horos, version 1.1.7) by reviewer 2 and reviewer 3 for inter-observer reliability assessment. The second reviewer repeated the measurements in the iNAV dataset twice, to assess intra-reviewer reliability.

### Statistical analysis

A Kolmogorov–Smirnov test was performed to test the null hypothesis that each variable is normally distributed at the 5% significance level. Data with normal distribution are presented as mean (± standard deviation) and compared using a paired Wilcoxon signed-rank test. Data with non-normal distribution are described in median and interquartile range and compared using a Mann–Whitney U-test. Bland–Altman plots were used to assess agreement of measurements of corresponding segments using both imaging techniques and intra-rater agreement for the proposed iNAV T2-prepared bSSFP. Intra-class correlation coefficient was applied to determine the inter- and intra-reviewer concordance. Intraclass correlation estimates and their 95% confident intervals were calculated, based on two-way mixed effects model to define the inter- and intra-reviewer reliability.

Statistical analysis was performed using SPSS (version 26.0.0.1, Statistical Package for the Social Sciences, International Business Machines, Inc., Armonk, New York, USA) and Prism Graphpad (Version 9.1.0). For all tests the statistically significant difference was set to P < 0.05.

## Results

Both imaging sequences were completed successfully in all 35 subjects (36 ± 13 years, range 18–66 years; 14 female). Additional file 1: Table S1, presents the patient cohort with the corresponding diagnoses. The scan time for the iNAV acquisition was on average 3.1 ± 0.5 min vs. 12.0 ± 3.0 min for the clinical dNAV sequence (p = 0.005). The reconstruction time for the proposed iNAV sequence was 3 ± 0.3 min in the scanner.

### Visual comparison of the proposed iNAV and the conventional dNAV aortic CMRA

**Fig. 2:**
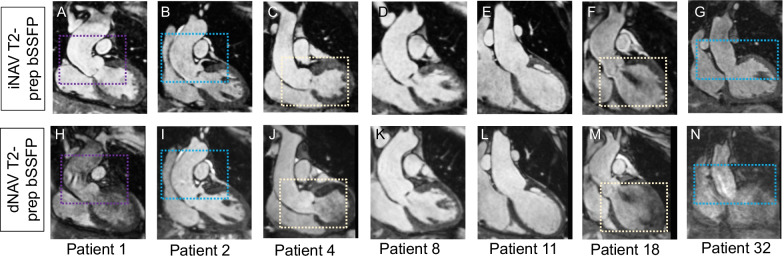
Coronal images of the aortic root and ascending aorta for seven representative patients. Proposed (iNAV) and conventional (dNAV) non-contrast enhanced CMRA are showed in the first and second row respectively. Remaining respiratory motion artefacts are less noticeable with the proposed approach (blue boxes). Signal homogeneity in the blood pool is significantly improved with the proposed approach in the aortic root and ascending aorta (purple boxes) and left ventricle (yellow boxes)

**Fig. 3:**
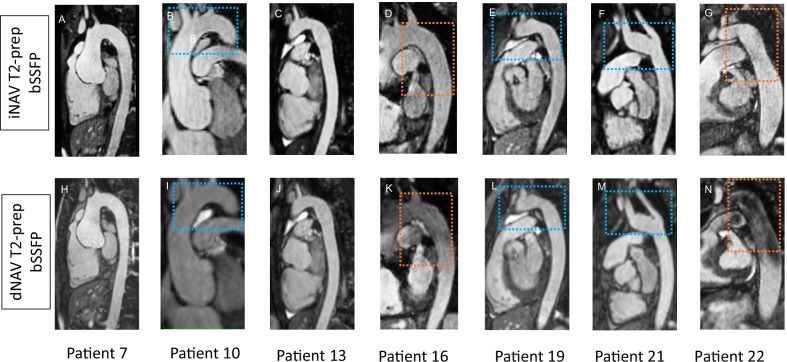
Sagittal images of the transverse arch and descending aorta for seven representative patients. Proposed (iNAV) and conventional (dNAV) non-contrast enhanced CMRA are showed in the first and second row respectively. Remaining respiratory motion artefacts are less noticeable with the proposed approach (blue boxes). Signal homogeneity in the blood pool is significantly improved with the proposed approach in the aortic arch and descending aorta (orange boxes)

Figures 2 and 3 show visual comparisons between the iNAV-based approach and the conventional dNAV-based acquisition for 7 representative subjects, focusing on the aortic root and ascending aorta, and the transverse arch and descending aorta, respectively. Blurring from residual respiratory motion is attenuated with the proposed approach along with flow-related artefacts. The proposed approach generates robust aortic imaging in patients with high body mass index (BMI), irregular breathing pattern and in one patient with atrial fibrillation; Additional file 2: Fig. S1, and Additional file 3: Fig. S2.

### Image quality scores comparison for iNAV versus dNAV aortic CMRA

Overall diagnostic confidence was higher for the proposed iNAV sequence versus the clinical dNAV sequence for all three reviewers, although it did not reach statistical significance (Reviewer 1: 3.9 ± 0.3 vs. 3.8 ± 0.4, P = 0.7; Reviewer 2: 4.0 ± 0.2 vs. 3.9 ± 0.3, P = 0.4; Reviewer 3: 3.8 ± 0.4 vs. 3.7 ± 0.6, P = 0.3).

### Qualitative image quality analysis results

**Fig. 4:**
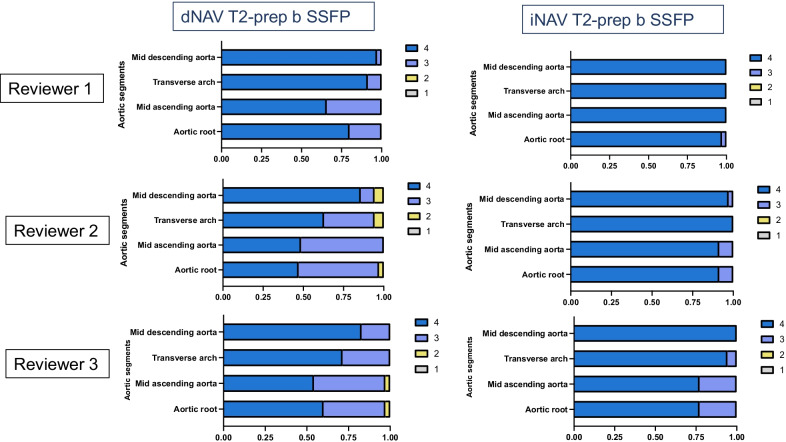
Image quality scores with respect to remaining artefacts/blurring from respiratory motion. Image quality scores (1: severe artefact to 4: minimal artefact from respiratory motion) with respect to remaining artefacts/blurring from respiratory motion for the proposed iNAV (right) in comparison to the clinical dNAV (left) CMRA sequence for the three reviewers for the mid descending aorta, mid aortic arch, mid ascending aorta and aortic root

**Fig. 5:**
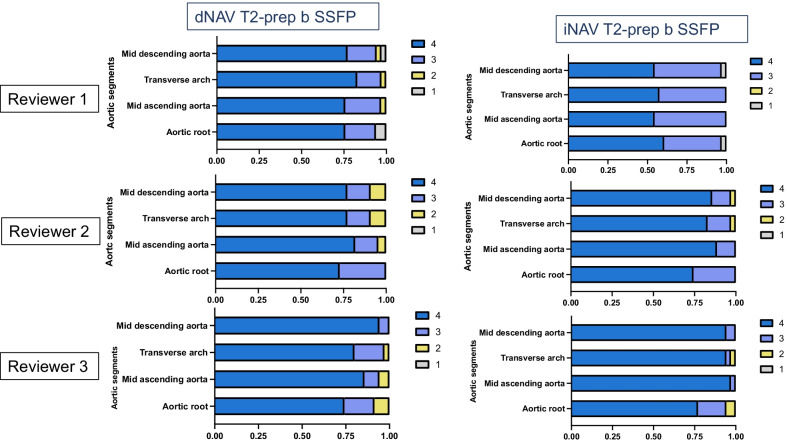
Image quality scores comparison with respect to homogeneity of blood signal. Image quality scores (1: non-diagnostic to 4: excellent) with respect to homogeneity of blood signal for the proposed iNAV (right) in comparison to the clinical dNAV (left) CMRA sequence for the three reviewers for the mid descending aorta, mid aortic arch, mid ascending aorta and aortic root

Image quality scores with respect to blurring from respiratory motion and homogeneity of blood signal intensity for the proposed iNAV in comparison to the clinical dNAV sequence are shown in Figs. 4 and 5 and Table 1. Additional file 4: Figure S3 includes example cases of different image quality visual assessment scores by the most senior expert cardiologist. Comparison between the dNAV and the iNAV approach shows that the proposed iNAV method is superior or similar to the conventional with regards to blurring from respiratory motion. In particular, two reviewers graded the image quality scores of the ARoot as superior for the proposed approach [Reviewer 1: 4 (3, 4) vs. 4 4, 4), p = 0.05 and Reviewer 2: 3 (3, 4) vs. 4 (4, 4), p < 0.001], whereas for one reviewer the image quality scores were similar [Reviewer 3: 4 (3, 4) vs. 4 (4, 4), p = 0.15]. The corresponding analysis showed significant higher scores for all reviewers for the proposed approach in the mAA [Reviewer 1: 4 (3, 4) vs. 4 (4, 4), p < 0.001, Reviewer 2: 3 (3, 4) vs. 4 (4, 4) p < 0.001, Reviewer 3: 4 (3, 4) vs. 4 (4, 4), p = 0.05] and for two of the reviewers for the mAAr [Reviewer 2: 4 (3, 4) vs. 4 (4, 4), p = 0.002 and Reviewer 3: 4 (3, 4) vs. 4 (4, 4) vs. p = 0.02], whereas for one of the reviewers the corresponding image quality scores were similar for the respective segment [mAAr Reviewer 1: 4 (4, 4) vs. 4 (4, 4), p = 0.24]. The image quality scores for the mDA were comparable between the two methods for the three reviewers [4 (4, 4) vs. 4 (4, 4), p = 1; 4 (4, 4) vs. 4 (4, 4), p = 0.1; 4 (4, 4) vs. 4 (4, 4), p = 0.1].

**Table 1:** Comparison of the image quality scores between the dNAV and the iNAV T2-prepared bSSFP sequences

Structure	Blurring from respiratory motion		Signal homogeneity in the blood pool	
	dNAV	iNAV	dNAV	iNAV
Aortic root (Reviewer 1)	(25th percentile, 75th percentile) 4 (3, 4), p = 0.05*	(25th percentile, 75th percentile) 4 (4, 4)	(25th percentile, 75th percentile) 4 (3, 4), p = 0.29	(25th percentile, 75th percentile) 4 (3, 4)
Aortic root (Reviewer 2)	3 (3, 4), p < 0.001*	4 (4, 4)	4 (3, 4), p = 0.8	4 (3, 4)
Aortic root (Reviewer 3)	4 (3, 4), p = 0.15	4 (4, 4)	4 (3, 4), p = 0.8	4 (4, 4)
Mid ascending aorta (Reviewer 1)	4 (3, 4), p < 0.001*	4 (4, 4)	4 (3, 4), p = 0.12	4 (3, 4)
Mid ascending aorta (Reviewer 2)	3 (3, 4), p < 0.001*	4 (4, 4)	4 (3, 4), p = 0.17	4 (4, 4)
Mid ascending aorta (Reviewer 3)	4 (3, 4), p = 0.05*	4 (4, 4)	4 (4, 4), p = 0.14	4 (4, 4)
Mid aortic arch (Reviewer 1)	4 (4, 4) p = 0.24	4 (4, 4)	4 (4, 4), p = 0.06	4 (3, 4)
Mid aortic arch (Reviewer 2)	4 (3, 4), p = 0.002*	4 (4, 4)	3.75 (4, 4), p = 1	4 (3, 4)
Mid aortic arch (Reviewer 3)	4 (3, 4), p = 0.02*	4(4,4)	4(4, 4), p = 0.2	4 (4, 4)
Mid descending aorta (Reviewer 1)	4 (4, 4), p = 1	4 (4, 4)	4 (3, 4), p = 0.1	4 (3, 4)
Mid descending aorta (Reviewer 2)	4 (4, 4), p = 0.1	4 (4, 4)	4 (4, 4), p = 0.5	4 (4, 4)
Mid descending aorta (Reviewer 3)	4 (4, 4), p = 0.1	4 (4, 4)	4 (4, 4), p = 1	4 (4, 4)

Comparison of the image quality scores with respect to blurring from respiratory motion and signal homogeneity in the blood pool for the three reviewers between the diaphragmatic navigator (dNAV) and the image based navigator (iNAV) T2-prepared balanced steady state free precession (bSSFP) sequences. The evaluation was performed at the level of the aortic root, mid ascending aorta, mid aortic arch and mid descending aorta. Asterisks (*) note the pairs with statistically significant difference in the image quality scores

Comparison between the dNAV and iNAV CMRA image quality scores with respect to signal homogeneity in the blood pool, showed similar scores for all three reviewers in the ARoot, mAA, mAAr and mDA. Inhomogeneity in the blood signal leading to non-diagnostic images was noted in 2 segments in the research sequence versus 5 for the clinical sequence for reviewer 1; 2 segments (iNAV) versus 6 (dNAV) for reviewer 2 and 3 segments (iNAV) versus 6 (dNAV) for reviewer 3.

### Quantitative image quality analysis results

The results demonstrate no statistically significant difference in CR in the iNAV images versus the dNAV images of the mAA. There is statistically significant higher CR of the dNAV images versus the iNAV images at the level of ARoot, the mAAr and mDA. The proposed sequence results in statistically significant improved signal homogeneity when the RSD scores (four circular ROIs) are averaged along the entire thoracic aorta [6.5 (4.6, 8.6) vs. 5.1 (4.4, 6.5), p = 0.002]. The signal homogeneity was superior in the research sequence in the mAA and not statistically significantly different between the two methods for the remaining three segments. These results are summarized in Table 2 and Additional file 5: Fig. S4.

**Table 2:** Comparison of the contrast ratio (CR) and relative standard deviation (RSD) signal intensity

Structures	CR dNAV	CR iNAV	Relative standard deviation (RSD) signal intensity dNAV	Relative standard deviation (RSD) signal intensity iNAV
Aortic root	2 (1.5, 2.4), p = 0.001*	1.5 (1.2, 1.7)	5.9 (4.2, 8.3), p = 0.8	5.6 (4.7, 6.6)
Mid ascending aorta	1.9 (1.4, 2.2), p = 0.16	1.5 (1.3, 1.9)	5.2 (3.9, 7.9), p = 0.05*	4.7 (2.5, 6.1)
Mid aortic arch	1.9 (1.2, 2.3), p = 0.006*	1.3 (1, 1.5)	6.2 (3.7, 8.4), p = 0.1	4.4 (3.8, 5.7)
Mid descending aorta	2.1 (1.5, 2.4), p < 0.001*	1.1 (1, 1.5)	5.1 (3.7, 7.4), p = 0.6	5.7 (6.8, 4.4)
Whole aorta			6.5 (4.6, 8.6), p = 0.002*	5.1 (4.4, 6.5)

Comparison of the contrast ratio (CR) and relative standard deviation (RSD) signal intensity between the dNAV T2-prepared bSSFP and the iNAV T2-prepared bSSFP CMRA in four aortic segments and the whole thoracic aorta

Asterisks (*) note the pairs with statistically significant difference in the contrast ratio and RSD of the signal intensity

### Co-axial aortic diameter measurements with iNAV versus dNAV CMRA

**Fig. 6:**
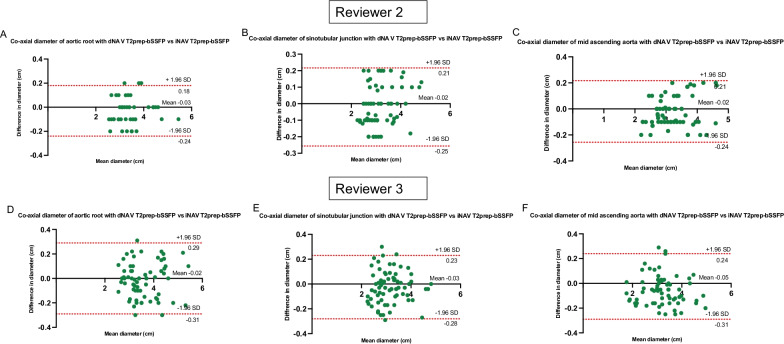
Bland–Altman plots for co-axial aortic dimensions between the dNAV and the iNAV T2prepared-bSSFP. Bland–Altman plots for co-axial diameter measurements of the aortic root, sinotubular unction and mid ascending aorta between the dNAV and the iNAV T2prepared-bSSFP sequence for reviewer 2 (**A**–**C**) and reviewer 3 (**D**–**F**). The black line indicates the mean bias of the diameter measurements whereas the red lines represent the 95% confidence interval. Values are given in cm. **A** Good agreement with a mean difference between methods of − 0.03 cm for reviewer 2 for the aortic root (95% CI − 0.24 to 0.18); **B** good agreement with a mean difference of − 0.02 cm for reviewer 2 for the sinotubular junction (95% CI − 0.25 to 0.21); **C** good agreement with a mean difference of − 0.02 cm for reviewer 2 for the mid ascending aorta (95% CI − 0.24 to 0.21); **D** good agreement with a mean difference of − 0.02 cm for reviewer 3 for the aortic root (95% CI − 0.31 to 0.29); **E** good agreement with a mean difference of − 0.03 cm for the sinotubular junction for reviewer 3 (95% CI − 0.28 to 0.23); **F** good agreement with a mean difference of − 0.05 cm for reviewer 3 for the mid ascending aorta (95% CI − 0.31 to 0.24)

Co-axial diameter measurements performed using iNAV and dNAV CMRA were compared using Bland–Altman analysis for the ARoot, STJ, mAA, mAAr, mDA, dDA for two of the reviewers (Fig. 6 and Additional file 6: Fig. S5). The results demonstrate good agreement with a mean difference between methods of less than 0.05 cm for both reviewers. Mean bias and upper and lower limits of agreement are cited in detail in Table 3.

**Table 3:** Mean bias and upper and lower limits of agreement for co-axial aortic diameter measurements

	Mean Bias	Upper limit of agreement (+ 1.96 SD)	Lower limit of agreement (− 1.96 SD)
Reviewer 2			
Aortic root	− 0.03	0.18	− 0.24
Sinotubular junction	− 0.02	0.21	− 0.25
Mid ascending aorta	− 0.02	0.21	− 0.24
Mid aortic arch	0.003	0.2	− 0.21
Mid descending aorta	− 0.02	0.15	− 0.2
Distal descending aorta	0.03	0.2	− 0.13
Reviewer 3			
Aortic root	− 0.02	0.29	− 0.31
Sinotubular junction	− 0.03	0.23	− 0.28
Mid ascending aorta	− 0.05	0.24	− 0.31
Mid aortic arch	0.01	0.33	− 0.31
Mid descending aorta	0.006	0.29	− 0.25
Distal descending aorta	− 0.02	0.2	− 0.25

Mean bias and upper and lower limits of agreement for co-axial aortic diameter measurements with iNAV versus dNAV approach for two reviewers

### Inter- and intra-reviewer agreement

There was excellent inter-reviewer agreement for the iNAV images in the co-axial diameter measurements of the ARoot, STJ, mAA, mAAr, mDA and dDA. Results are shown in Table 4. There was excellent intra-reviewer agreement for the iNAV T2-prepared bSSFP in the co-axial diameter measurements of the corresponding aortic landmarks. Results are shown in Table 5 and corresponding Bland–Altman plots in Additional file 7: Fig. S6.

**Table 4:** Intraclass correlation coefficient (ICC) for inter-reviewer and intra-reviewer agreement and 95% confidence interval (CI)

Structure	Inter-observer ICC (95% CI)	Intra-observer ICC (95% CI)
Aortic root	0.9 (0.85, 0.94)	0.99 (0.99, 1)
Sinotubular junction	0.89 (0.83, 0.93)	0.99 (0.99, 1)
Mid ascending aorta	0.88 (0.82, 0.94)	0.99 (0.99, 1)
Mid aortic arch	0.97 (0.95, 0.98)	0.99 (0.98, 0.99)
Mid descending	0.91 (0.85, 0.94)	0.97 (0.98, 0.99)
Distal descending aorta	0.94 (0.92, 0.99)	0.98 (0.99, 0.99)

Intraclass correlation coefficient (ICC) for inter-reviewer and intra-reviewer agreement and 95% confidence interval (CI)

**Table 5:** Mean bias and upper and lower limits of agreement for intra-rater co-axial aortic diameter measurements

Intra-rater agreement	Mean bias	Upper limit of agreement (+ 1.96 SD)	Lower limit of agreement (− 1.96 SD)
Aortic root	0.002	0.13	− 0.13
Sinotubular junction	0.01	0.14	− 0.11
Mid ascending aorta	0.005	0.15	− 0.14
Mid aortic arch	0.02	0.15	− 0.11
Mid descending aorta	0.01	0.16	− 0.14
Distal descending aorta	− 0.007	0.12	− 0.15

Mean bias and upper and lower limits of agreement for intra-rater co-axial aortic diameter measurements with the iNAV approach

## Discussion

This work describes the evaluation of a prototype iNAV-based motion-corrected 3D CMRA sequence in patients with thoracic aortic disease for dimensioning and imaging of the thoracic aorta versus the clinical standard dNAV gated sequence. The main findings of our study are that the proposed iNAV sequence (1) produces good quality imaging of the thoracic aorta in a faster 3-min scan with a 3-min ***inline*** reconstruction; (2) generates 3D images with homogenous blood pool signal along the entire aorta; (3) introduces non-rigid respiratory motion correction taking into account the differences in motion between the arch, ascending and descending aorta; (4) provides reliable quantification of vessel dimensions, achieving good agreement with the clinical standard, (5) shows excellent inter and intra-observer agreement in diameter measurements. The overall diagnostic confidence is comparable between the proposed and the clinical methods, although non-diagnostic images due to signal inhomogeneity are less frequently observed with the proposed method. In comparison to the conventional approach, the proposed sequence provided significantly faster and more predictable acquisition times along with superior quality images with respect to remaining respiratory motion artefacts.

The respiratory motion correction strategies affect both the scan time and the conspicuity of the reconstructed images. The conventional dNAV gating leads to prolonged (usually by a factor of two or more) and unpredictable scan times since data acquired outside a pre-defined respiratory window (usually end-expiration) is rejected and need to be reacquired at a later time [24]. The proposed iNAV approach does not rely on an operator’s expertise to properly define navigator gating and achieves 100% respiratory scan efficiency, contributing to shorter and more consistent scan times. Additionally, in conventional dNAV gating, the respiratory-induced motion of the aorta is estimated indirectly from the right hemi-diaphragmatic displacement using a gating window of 7 mm, with a slice tracking of 0.6, that corresponds only to the FH cardiac displacement. It has been previously shown that the optimal estimation of aortic displacement varies across the segments of the thoracic aorta, the vertical and the horizontal axes and also for each subject with different underlying disease, implying that motion artifacts may not be resolved fully if a fixed factor for all parts of the aorta, corresponding solely to the FH motion correction is used [29, 30, 37]; introducing residual motion blurring. Studies in diverse patient groups have shown that respiratory-induced displacement of the aortic arch differs along its segments and presents greater variability post graft interposition, which could be relevant for a subgroup of this patient cohort, rendering the non-rigid motion correction an important parameter for optimised thoracic aortic imaging [37, 38].

The blood signal in the iNAV datasets proved to be more uniform, with reduced standard deviation of the signal intensity across all aortic segments. Although the visual comparison assessment did not show any significant difference for two of the reviewers, a higher interquartile range can be observed in the clinical sequence in the quantitative measurement at the aortic segments. For the total thoracic aorta, the signal intensity is statistically significant more homogenous with the proposed sequence. Additionally, inhomogeneity in the blood signal leading to non-diagnostic images was noted in more segments for the clinical sequence compared to the proposed for all three reviewers. The signal uniformity in the entire thoracic aorta is potentially attributed to the four composite refocusing pulses applied in the T2 preparation module for the proposed sequence (MLEV4) compared to the clinical sequence (MLEV2), that mitigate artefacts due to flow in the bSSFP signal [39] along with the more sophisticated motion correction proposed (translational and non-rigid).

The improved CR of the dNAV approach in the majority of aortic segments was most likely obtained because of the larger number of slices required for acquisition in the sagittal orientation. The proposed iNAV approach (coronal orientation) requires fewer (72–88) slices to cover the entire aorta than the dNAV approach which was acquired in sagittal orientation (as per guidelines in our clinical services), thus resulting in lower signal to noise for the iNAV approach. However, coronal orientation enables simpler radiographic planning and evaluation of the subclavian arteries through their entire thoracic course (Additional file 8: Fig. S7). It is also possible that the diaphragmatic navigator may result in a more effective T2-preparation, producing a higher aortic blood pool/myocardium CR. This is however hard to prove at this point, as the start-up profiles that are used with the iNAV approach to encode the motion are also applied to catalyse the magnetisation towards the steady state. Additionally, the iNAV is implemented with a high–low profile order acquiring the centre of k-space in close temporal proximity to the 3D imaging sequence, which might potentially offset the proximity of the T2 preparation pulse to the image acquisition with the diaphragmatic navigator. However, the threefold acceleration, versus the two-fold in the clinical sequence, may also contribute to the lower aortic blood pool/myocardium contrast ratio in our proposed sequence. Lastly, the reordering of the k-space is linear for the clinical sequence, whereas the iNAV approach utilises a variable-density Cartesian trajectory with spiral-like order (VD-CASPR). The clinical evaluation showed that the proposed sequence is superior or comparable to the clinical, in decision-making and diagnostic confidence, despite the difference in this particular quality metric.

Previous approaches, including stack-of-stars [40] and radial [22, 41] k-space sampling with self-navigation of respiratory motion have been proposed for aortic CMRA, albeit with longer total acquisition times (average duration of 6 min and 13 min for spatial resolution of 1.5 mm^3^ and 1.1 mm^3^, respectively), compared to our sequence. Furthermore, the Golden-angle RAdial Sparse Parallel imaging (GRASP) reconstruction framework adopted for the stack of stars sampling and the GRASP with eXtra Dimensions (XD-GRASP) applied in radial k-space sampling [22, 40], necessitate offline reconstruction with reported duration of 4.7 h and 15–30 min for the corresponding studies, which may hinder their immediate clinical adoption.

The proposed efficient aortic CMRA framework could further benefit clinical practice as it can be gauged as a potential alternative to avoid application of ionising radiation, which is important in serial follow-up of young patients, and also obviates need for intravenous contrast injection compared to both CTA and CE-CMRA, thus reducing preparation time and costs. The fast acquisition time in concert with inline reconstruction contributes further to clinical efficiency and cost reduction.

## Limitations

We performed a single-centre study with a limited number of patients. However, as the observed inter-method bias for maximum thoracic aorta diameter between the clinical dNAV CMRA and the proposed iNAV CMRA approach was small with narrow limits of agreement, we consider the sample size sufficient for showing non-inferiority of iNAV CMRA. Further assessment of precision, through reproducibility studies between vendors and sites, should be investigated. The proposed sequence provided high quality CMRA in an acquisition during atrial fibrillation (Additional file 3: Fig. S2). An extensive validation will need to be performed in a multi-center study recruiting a larger patient cohort with different underlying rhythm disorders.

This study did not compare the proposed approach to a CE dNAV-based CMRA because our clinical practice favours NC acquisitions. We did not perform comparison to CTA either, in view of the current institutional guidelines, suggesting CMRA for non-acute thoracic aortic disease. Both CTA and CMRA are commonly used for aortic imaging and the current American Heart Association and European Society of Cardiology guidelines do not specify a preferred imaging modality for the assessment of non-emergent aortic disease [1, 3]. CTA has substantial drawbacks, including exposure to ionising radiation and iodinated contrast [1, 42], that are obviated with CMRA. Additionally, the sensitivity and specificity of CMRA has been shown to be equivalent to or may exceed those for CTA, and therefore CMRA is frequently preferred for patients with aortopathies, who return for follow-up examinations at frequent short-term intervals [17].

## Future directions

A direct comparison between the application of conventional respiratory navigator with non -rigid motion correction and the same threefold acceleration factor with variable density pattern versus the proposed sequence will be explored in future work, to allow for better understanding of the sole contribution of image-based navigation to image quality, respiratory artifact, and inhomogeneity changes in the CMRA.

The homogeneous blood pool along the entire aorta, as obtained with the proposed sequence (Figs. 2, 3, Additional file 2: Fig. S1, Additional file 3: Fig. S2 and Additional file 9: Fig. S8), can be of particular interest in paediatric and adult congenital heart disease patients because it allows the examination of all intrapericardiac and aortic structures for an efficient anatomical assessment. The rapid acquisition achieved by the proposed method may limit patients’ discomfort, which is relevant especially for children. This will be investigated in future studies.

## Conclusions

This study describes the performance of an accelerated, free breathing, NC ECG‐triggered, thoracic CMRA approach with Cartesian sampling and image-based non-rigid respiratory motion correction. The proposed approach produces good image quality at isotropic spatial resolution (1.6 mm^3^) in fast acquisition (3 min) and in-line reconstruction time (3 min) facilitating adoption in clinical routine and reduction in imaging costs. Blurring from respiratory motion was mitigated with the proposed approach along with improved signal homogeneity. The overall diagnostic confidence was not significantly different between the clinical and the proposed approach, and the mean vessel diameters were in good agreement with excellent intra and inter-observer reproducibility.

## Supplementary Information

**Additional file 1: Table S1.** Patient cohort with the corresponding baseline characteristics. Patient cohort with the corresponding baseline characteristics

**Additional file 2: Figure S1.** Transverse and coronal views of the aorta for five patients with challenging acquisition. Robust aortic imaging with the iNAV T2prepared-bSSFP in patients with high BMI (Patient 24: A & F, Patient 27: B & G, Patient 29: C & H, Patient 32: E & J). Flow (red arrows) and respiratory-related (yellow arrows) artefacts are diminished. Significant attenuation of the respiratory motion artefacts in two patients with highly irregular breathing pattern; green arrows (Patient 30, D & I; Patient 32: E & J).

**Additional file 3: Figure S2.** Sagittal and coronal views of the aorta for three patients with challenging acquisition. Robust aortic imaging with the iNAV T2prep-bSSFP in a patient with atrial fibrillation during the scan (Patient 26: A & D), in one patient with high BMI (Patient 27, B & E) and in one patient with highly irregular breathing pattern (Patient 30, C & F). Significant flow-related artefacts and blurring from respiratory motion (arrowheads) are alleviated.

**Additional file 4: Figure S3.** Example cases of different image quality visual assessment scores reported by an expert cardiologist. Example cases of different image quality visual assessment scores reported by an expert cardiologist with regards to blurring from respiratory motion (4 = severe artefact, 2 = significant artefact, 3 = mild artefact, and 4 = minimal artefact) and homogeneity of blood signal intensity (1 = non diagnostic, 2 = poor, 3 = adequate-good, 4 = excellent).

**Additional file 5: Figure S4.** Relative standard deviation of signal intensity quantification in the whole thoracic aorta. Relative standard deviation of signal intensity quantification in the whole thoracic aorta for the dNAV T2-prep bSSFP and the iNAV T2-prep bSSFP.

**Additional file 6: Figure S5.** Bland–Altman plots for co-axial diameter measurements of mid aortic arch, mid descending aorta and distal descending aorta between the dNAV and the iNAV T2prep-bSSFP sequence for reviewer 2 (A, B, C) and reviewer 3 (D, E, F). Bland–Altman plots for co-axial diameter measurements of the mid aortic arch, mid descending aorta and distal descending aorta between the dNAV and the iNAV T2prep-bSSFP sequence for reviewer 2 (A, B, C) and reviewer 3 (D, E, F). The black line indicates the mean bias of the diameter measurements whereas the red lines represent the 95% confidence interval. Values are given in cm. **A** Good agreement with a mean difference 0.003 cm for reviewer 2 for the mid aortic arch (95% CI − 0.21 to 0.2) for reviewer 2; **B** good agreement with a mean difference of − 0.02 cm for reviewer 2 for the mid descending aorta (95% CI − 0.2 to 0.15); **C** good agreement with a mean difference of 0.03 cm for reviewer 2 for the distal descending aorta (95% CI − 0.13 to 0.2); **D** good agreement with a mean difference of 0.01 cm for reviewer 3 for the mid aortic arch arch (95% CI − 0.31 to 0.33); **E** good agreement with a mean difference of 0.006 cm for reviewer 3 for the mid descending aorta (95% CI − 0.25 to 0.29); **F** good agreement with a mean difference of − 0.02 cm for reviewer 3 for the distal descending aorta (95% CI − 0.25 to 0.2).

**Additional file 7: Figure S6.** Bland–Altman plots for intra-rater co-axial aortic diameter measurements with the iNAV T2prep-bSSFP sequence for reviewer 2. Bland–Altman plots for intra-rater co-axial diameter measurements of the aortic root, sinotubular unction, mid ascending aorta, mid aortic arch, mid and distal descending aorta with the iNAV T2prep-bSSFP sequence for reviewer 2. The black line indicates the mean bias of the diameter measurements whereas the red lines represent the 95% confidence interval. Values are given in cm. **A** Good agreement with a mean difference between methods of − 0.002 cm for the aortic root (95% CI − 0.13 to 0.13); **B** good agreement with a mean difference of 0.01 cm for the sinotubular junction (95% CI − 0.11 to 0.14); **C** good agreement with a mean difference of 0.005 cm for the mid ascending aorta (95% CI − 0.14 to 0.15); **D** good agreement with a mean difference of 0.02 cm for the mid aortic arch (95% CI − 0.11 to 0.15); **E** Good agreement with a mean difference of 0.01 cm for the mid descending aorta (95% CI − 0.14 to 0.16); **F** good agreement with a mean difference of − 0.007 cm for the distal descending aorta (95% CI − 0.15 to 0.12).

**Additional file 8: Figure S7.** Coronal visualisation of the whole chest and the thoracic vasculature. Coronal orientation in planning allows visualisation of the subclavian arteries and potential aneurysms or stenosis along their entire thoracic course.

**Additional file 9: Figure S8.** Segmental depiction of the intracardiac anatomy and the vessels connected to the heart. Segmental depiction of the pulmonary veins, the intracardiac anatomy, great arteries and coronary arteries.

## Data Availability

The datasets used and analysed during the current study are available from the corresponding author upon reasonable request.

## Abbreviations

1D: One dimensional
2D: Two dimensional
3D: Three dimensional
Aroot: Aortic root
BMI: Body mass index
bSSFP: Balanced steady state free precession
CE: Contrast enhanced
CMR: Cardiovascular magnetic resonance
CMRA: Cardiovascular magnetic resonance angiography
CR: Contrast ratio
CTA: Computed tomography angiography
dDA: Distal descending aorta
dNAV: Diaphragmatic-navigator
ECG: Electrocardiogram
FH: Foot head
FOV: Field-of-view
GRASP: Golden-angle RAdial Sparse Parallel imaging
iNAV: Image-based navigator
mAA: Mid ascending aorta
mAAr: Mid aortic arch
mDA: Mid descending aorta
MLEV2: Malcolm-Levitt phase cycling
NC: Non-contrast
RL: Right-left
ROI: Region-of-interest
RSD: Relative standard deviation
SENSE: Sensitivity Encoding
SI: Signal intensity
STJ: Sinotubular junction
TE: Echo time
TR: Repetition time
XD-GRASP: GRASP with eXtra Dimensions

## References

[1] Hiratzka LF, Bakris GL, Beckman JA (2010). 2010 ACCF/AHA/AATS/ACR/ASA/SCA/SCAI/SIR/STS/SVM guidelines for the diagnosis and management of patients with thoracic aortic disease: a report of the American college of cardiology foundation/American heart association task force on practice guidelines, American association for thoracic surgery, American college of radiology, American stroke association, society of cardiovascular anesthesiologists, society for cardiovascular angiography and interventions, society of interventional radiology, soc. Circulation.

[2] Yoshioka K, Tanaka R (2010). MRI and MRA of aortic disease. Ann Vasc Dis.

[3] Erbel R, Aboyans V, Boileau C (2014). 2014 ESC guidelines on the diagnosis and treatment of aortic diseases: document covering acute and chronic aortic diseases of the thoracic and abdominal aorta of the adult. The Task Force for the Diagnosis and Treatment of Aortic Diseases of the European Society of Cardiology (ESC). Eur Heart J.

[4] Pastry P, Hughes V, Hayes P (2015). The ETTAA study protocol: a UK-wide observational study of ‘effective treatments for thoracic aortic aneurysm’. BMJ Open.

[5] Go AS, Mozaffarian D, Roger VL (2013). Heart disease and stroke statistics–2013 update: a report from the American Heart Association. Circulation.

[6] von Kodolitsch Y, Rybczynski M, Detter C, Robinson PN (2008). Diagnosis and management of Marfan syndrome. Future Cardiol.

[7] Russo V, Renzulli M, La Palombara C, Fattori R (2006). Congenital diseases of the thoracic aorta. Role of MRI and MRA. Eur Radiol.

[8] Prince MR, Narasimham DL, Jacoby WT, Williams DM, Cho KJ, Marx MV, Deeb GM (1996). Three-dimensional gadolinium-enhanced MR angiography of the thoracic aorta. AJR Am J Roentgenol.

[9] Cesare ED, Giordano AV, Cerone G, De Remigis F, Deusanio G, Masciocchi C (2000). Comparative evaluation of TEE, conventional MRI and contrast-enhanced 3D breath-hold MRA in the post-operative follow-up of dissecting aneurysms. Int J Card Imaging.

[10] Galizia MS, Febbo JA, Popescu AR (2012). Steady state imaging of the thoracic vasculature using inversion recovery FLASH and SSFP with a blood pool contrast agent. J Cardiovasc Magn Reson.

[11] Guo BJ, Yang ZL, Zhang LJ (2018). Gadolinium deposition in brain: current scientific evidence and future perspectives. Front Mol Neurosci.

[12] Weinreb J, Rodby R, Yee J (2021). Use of intravenous gadolinium-based contrast media in patients with kidney disease: consensus statements from the American college of radiology and the national kidney foundation. Kidney Med.

[13] Gebker R, Gomaa O, Schnackenburg B, Rebakowski J, Fleck E, Nagel EE (2007). Comparison of different MRI techniques for the assessment of thoracic aortic pathology: 3D contrast enhanced MR angiography, turbo spin echo and balanced steady state free precession. Int J Cardiovasc Imaging.

[14] Naehle CP, Müller A, Willinek WA (2009). First-pass and steady-state magnetic resonance angiography of the thoracic vasculature using gadofosveset trisodium. J Magn Reson Imaging.

[15] Veldhoen S, Behzadi C, Lenz A (2017). Non-contrast MR angiography at 1.5 Tesla for aortic monitoring in Marfan patients after aortic root surgery. J Cardiovasc Magn Reson.

[16] Krishnam MS, Tomasian A, Malik S, Desphande V, Laub G, Ruehm SG (2010). Image quality and diagnostic accuracy of unenhanced SSFP MR angiography compared with conventional contrast-enhanced MR angiography for the assessment of thoracic aortic diseases. Eur Radiol.

[17] Groth M, Henes FO, Müllerleile K, Bannas P, Adam G, Regier M (2012). Accuracy of thoracic aortic measurements assessed by contrast enhanced and unenhanced magnetic resonance imaging. Eur J Radiol.

[18] Renker M, Varga-Szemes A, Schoepf UJ (2016). A non-contrast self-navigated 3-dimensional MR technique for aortic root and vascular access route assessment in the context of transcatheter aortic valve replacement: proof of concept. Eur Radiol.

[19] Snel GJH, Hernandez LM, Slart RH (2020). Validation of thoracic aortic dimensions on ECG-triggered SSFP as alternative to contrast-enhanced MRA. Eur Radiol.

[20] Bannas P, Groth M, Rybczynski M (2013). Assessment of aortic root dimensions in patients with suspected Marfan syndrome: intraindividual comparison of contrast-enhanced and non-contrast magnetic resonance angiography with echocardiography. Int J Cardiol.

[21] Aouad P, Jarvis KB, Botelho MF (2021). Aortic annular dimensions by non-contrast MRI using k-t accelerated 3D cine b-SSFP in pre-procedural assessment for transcatheter aortic valve implantation: a technical feasibility study. Int J Cardiovasc Imaging.

[22] Stroud RE, Piccini D, Schoepf UJ (2019). Correcting versus resolving respiratory motion in free-breathing whole-heart MRA: a comparison in patients with thoracic aortic disease. Eur Radiol Exp.

[23] Heerfordt J, Stuber M, Maillot A, Bianchi V, Piccini D (2020). A quantitative comparison between a navigated Cartesian and a self-navigated radial protocol from clinical studies for free-breathing 3D whole-heart bSSFP coronary MRA. Magn Reson Med.

[24] McConnell MV, Khasgiwala VC, Savord BJ (1997). Comparison of respiratory suppression methods and navigator locations for MR coronary angiography. AJR Am J Roentgenol.

[25] Stehning C, Börnert P, Nehrke K, Eggers H, Stuber M (2005). Free-breathing whole-heart coronary MRA with 3D radial SSFP and self-navigated image reconstruction. Magn Reson Med.

[26] Piccini D, Littmann A, Nielles-Vallespin S, Zenge MO (2012). Respiratory self-navigation for whole-heart bright-blood coronary MRI: methods for robust isolation and automatic segmentation of the blood pool. Magn Reson Med.

[27] Pamminger M, Kranewitter C, Kremser C (2021). Self-navigated versus navigator-gated 3D MRI sequence for non-enhanced aortic root measurement in transcatheter aortic valve implantation. Eur J Radiol.

[28] Sailer AM, Wagemans BAJM, Das M (2015). Quantification of respiratory movement of the aorta and side branches. J Endovasc Ther.

[29] Weber TF, Tetzlaff R, Rengier F (2009). Respiratory displacement of the thoracic aorta: physiological phenomenon with potential implications for thoracic endovascular repair. Cardiovasc Intervent Radiol.

[30] Suh GY, Beygui RE, Fleischmann D, Cheng CP (2014). Aortic arch vessel geometries and deformations in patients with thoracic aortic aneurysms and dissections. J Vasc Interv Radiol.

[31] Henningsson M, Koken P, Stehning C, Razavi R, Prieto C, Botnar RM (2012). Whole-heart coronary MR angiography with 2D self-navigated image reconstruction. Magn Reson Med.

[32] Addy NO, Ingle RR, Luo J (2017). 3D image-based navigators for coronary MR angiography. Magn Reson Med.

[33] Cruz G, Atkinson D, Henningsson M, Botnar RM, Prieto C (2017). Highly efficient nonrigid motion-corrected 3D whole-heart coronary vessel wall imaging. Magn Reson Med.

[34] Bustin A, Rashid I, Cruz G (2020). 3D whole-heart isotropic sub-millimeter resolution coronary magnetic resonance angiography with non-rigid motion-compensated PROST. J Cardiovasc Magn Reson.

[35] Jenista ER, Rehwald WG, Chen E (2013). Motion and flow insensitive adiabatic T2-preparation module for cardiac MR imaging at 3 tesla. Magn Resonan Med.

[36] Brittain JH, Hu BS, Wright GA, Meyer CH, Macovski A, Nishimura DG (1995). Coronary angiography with magnetization-prepared T2 contrast. Magn Reson Med.

[37] Suh GY, Fleischmann D, Beygui RE, Cheng CP (2017). Quantification of motion of the thoracic aorta after ascending aortic repair of type-A dissection. Int J Comput Assist Radiol Surg.

[38] Muhs BE, Vincken KL, van Prehn J, Stone MKC, Bartels LW, Prokop M (2006). Dynamic cine-CT angiography for the evaluation of the thoracic aorta; insight in dynamic changes with implications for thoracic endograft treatment. Eur J Vasc Endovasc Surg.

[39] Markl M, Alley MT, Elkins CJ, Pelc NJ (2003). Flow effects in balanced steady state free precession imaging. Magn Reson Med.

[40] Haji-Valizadeh H, Collins JD, Aouad PJ (2018). Accelerated, free-breathing, noncontrast, electrocardiograph-triggered, thoracic MR angiography with stack-of-stars k-space sampling and GRASP reconstruction. Magn Reson Med.

[41] Poskaite P, Pamminger M, Kranewitter C (2021). Self-navigated 3D whole-heart MRA for non-enhanced surveillance of thoracic aortic dilation: a comparison to CTA. Magn Reson Imaging.

[42] Wang C, Asch D, Cavallo J, et al. American College of Radiology Manual on Contrast Media. https://www.acr.org/-/media/ACR/files/clinical-resources/contrast_media.pdf. Accessed 11 Nov 2021.

