# Supplementary material for: Efficient non-contrast enhanced 3D Cartesian cardiovascular magnetic resonance angiography of the thoracic aorta in 3 min
Source: J Cardiovasc Magn Reson. 2022 Jan 10;24:5. doi: 10.1186/s12968-021-00839-9 (PMC8744314; doi:10.1186/s12968-021-00839-9)
Supplement: Supplementary file 1 — Additional file 1: Table S1. Patient cohort with the corresponding baseline characteristics. Patient cohort with the corresponding baseline characteristics [file 12968_2021_839_MOESM1_ESM.docx]

**Additional file 1: Table S1 Title: Patient cohort and corresponding baseline characteristics**

| **Identification details** | **Diagnosis & Procedures** | **Gender** | **Age** | **Height** | **Weight** | **BMI** | **Cardiac rhythm** |
| --- | --- | --- | --- | --- | --- | --- | --- |
| Patient 1 | Marfan’s syndrome. Aortic root aneurysm.  Aneurysm of the mid ascending aorta | M | 50 | 192 | 100 | 27.1 | Sinus rhythm |
| Patient 2 | Bicuspid aortic valve.  Aortic root dilatation.  Aneurysm of the proximal ascending aorta. | M | 46 | 175 | 87 | 28.4 | Sinus rhythm |
| Patient 3 | Coarctation of the aorta.  Dilatation of the proximal descending aorta.  Coarctation of the aorta repair. | M | 35 | 182 | 90 | 27.1 | Sinus rhythm |
| Patient 4 | Congenital aortic valve stenosis.  Moderate to severe aortic regurgitation post previous balloon valvuloplasty. | F | 32 | 155 | 55 | 22.9 | Sinus rhythm |
| Patient 5 | Congenital aortic valve stenosis. Distal ascending aortic aneurysm.  Valve sparing aortic root replacement with a 28mm Hemashield graft. | M | 40 | 178 | 120 | 37.9 | Sinus rhythm |
| Patient 6 | Bicuspid aortic valve with normal function. Tortuous aortic arch. Mild dilatation of the mid descending aorta . | F | 26 | 170 | 88 | 30.4 | Sinus rhythm |
| Patient 7 | Bicuspid aortic valve with fusion of the right and non-coronary cusp.  Dilatation of the mid ascending aorta | M | 55 | 180 | 83 | 25.6 | Sinus rhythm |
| Patient 8 | Congenital aortic stenosis.  Dilatation of the mid ascending aorta. | M | 19 | 167 | 73 | 26.2 | Sinus rhythm |
| Patient 9 | Coarctation of the aorta.  Normal dimensions of the thoracic aorta.  Coarctation of the aorta repair. | M | 32 | 172 | 90 | 30 | Sinus rhythm |
| Patient 10 | Bicuspid aortic valve with severe stenosis and moderate regurgitation. Aortic root aneurysm.  Dilated mid ascending aorta.    Aortic valve replacement with mechanical valve. | F | 36 | 181 | 91 | 27.8 | Sinus rhythm |
| Patient 11 | Bicuspid aortic valve with moderate stenosis.  Mid ascending aortic aneurysm.  Dilated transverse arch. | M | 33 | 182 | 72 | 21.7 | Sinus rhythm |
| Patient 12 | Bicuspid aortic valve . Aortic root aneurysm.  Dilated mid ascending aorta. | F | 29 | 187 | 109 | 31.2 | Sinus rhythm |
| Patient 13 | Congenital aortic valve stenosis. Coarctation of the aorta. Aberrant right subclavian artery.  Dilated mid ascending aorta.  Coarctation of the aorta repair. | M | 30 | 165 | 66 | 24.2 | Sinus rhythm |
| Patient 14 | Family history of aortic dissection. | F | 31 | 160 | 78 | 30 | Sinus rhythm |
| Patient 15 | Transposition of the great arteries. Aortic root aneurysm.  Dilated mid ascending aorta.  Arterial switch operation. | M | 30 | 182 | 74 | 22.3 | Sinus rhythm |
| Patient 16 | Bicuspid aortic valve. Mild stenosis.  Mild regurgitation  Dilated mid ascending aorta. | M | 27 | 182 | 86 | 25.6 | Sinus rhythm |
| Patient 17 | Marfan’s syndrome. Dilated aortic root. | F | 21 | 171 | 59 | 20 | Sinus rhythm |
| Patient 18 | Congenital aortic valve stenosis.  Aortic coarctation.  Aortic valvotomy and coarctation of the aorta repair. | F | 18 | 161 | 62 | 23.9 | Sinus rhythm |
| Patient 19 | Bicuspid aortic valve with mild stenosis. Aortic coarctation.  Coarctation of the aorta repair. | M | 56 | 169 | 68 | 23.8 | Sinus rhythm |
| Patient 20 | Bicuspid aortic valve.  Mild aortic regurgitation. | M | 21 | 180 | 64 | 19.8 | Sinus rhythm |
| Patient 21 | Functionally bicuspid aortic valve.  Mild aortic valve stenosis. | F | 28 | 163 | 94 | 35 | Sinus rhythm |
| Patient 22 | Congenital aortic valve stenosis. Dilated mid ascending aorta.  Mechanical aortic valve in situ with severe regurgitation. | F | 32 | 169 | 71 | 24.8 | Sinus rhythm |
| Patient 23 | Coarctation of the aorta  Bicuspid aortic valve  Aortic root dilatation  Stent in the aortic isthmus. | F | 44 | 150 | 70 | 31 | Sinus rhythm |
| Patient 24 | Dilated aortic root and ascending aorta  Restrictive perimembranous ventricular septal defect. | F | 27 | 185 | 103 | 30 | Sinus rhythm |
| Patient 25 | Dilated cardiomyopathy  Mild ascending aorta dilatation. | M | 46 | 171 | 74 | 25 | Sinus rhythm |
| Patient 26 | Mild aortic root and ascending aorta dilatation | M | 46 | 183 | 83 | 24.8 | Atrial fibrillation |
| Patient 27 | Ascending aorta aneurysm.  Hypertension. | M | 58 | 175 | 94 | 30.7 | Sinus rhythm |
| Patient 28 | Coarctation of the aorta.  Coarctation of the aorta repair. | F | 18 | 164 | 48 | 17.8 | Sinus rhythm |
| Patient 29 | Family history of aortic dissection.  Moderately dilated aortic root. | M | 66 | 177 | 109 | 34.8 | Sinus rhythm |
| Patient 30 | Moderately dilated ascending aorta.  Atrial septal defect. | F | 63 | 167 | 68 | 24.4 | Sinus rhythm |
| Patient 31 | Family history of aortic dissection.  Mildly dilated aortic root. | M | 18 | 180 | 73 | 22.5 | Sinus rhythm |
| Patient 32 | Aortic root aneurysm.  Moderately dilated ascending aorta. | M | 29 | 183 | 93 | 27.8 | Sinus rhythm |
| Patient 33 | Moderate to severe dilatation of the ascending aorta.  Aortic valve replacement with tissue valve. | M | 42 | 186 | 101 | 29.2 | Sinus rhythm |
| Patient 34 | Coarctation of the aorta.  Coarctation of the aorta repair. | F | 34 | 175 | 73 | 23.8 | Sinus rhythm |
| Patient 35 | Aortic root aneurysm  Severely dilated proximal ascending aorta.  Moderately dilated proximal descending aorta. | M | 39 | 193 | 86 | 23 | Sinus rhythm |

**Additional file 1: Table S1 legend:** Patient cohort and corresponding baseline characteristics
