# Supplementary figures and images for: Efficient non-contrast enhanced 3D Cartesian cardiovascular magnetic resonance angiography of the thoracic aorta in 3 min
Source: J Cardiovasc Magn Reson. 2022 Jan 10;24:5. doi: 10.1186/s12968-021-00839-9 (PMC8744314; doi:10.1186/s12968-021-00839-9)

## Slide 1
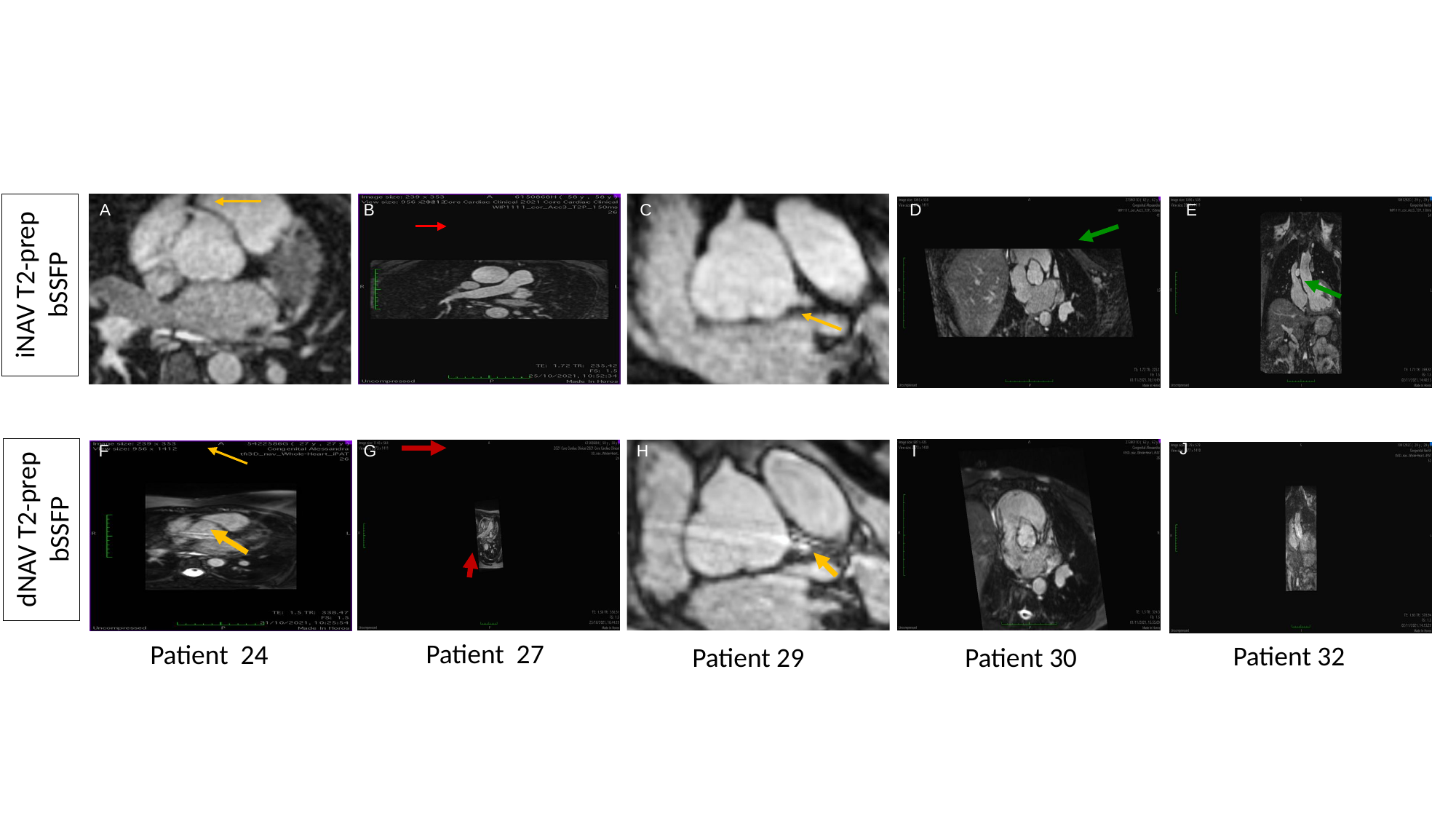

A
B
C
D
E
iNAV T2-prep bSSFP
J
F
G
H
I
dNAV T2-prep bSSFP
Patient 27
Patient 24
Patient 32
Patient 29
Patient 30

Supplement: Supplementary file 2 — Additional file 2: Figure S1. Transverse and coronal views of the aorta for five patients with challenging acquisition. Robust aortic imaging with the iNAV T2prepared-bSSFP in patients with high BMI (Patient 24: A & F, Patient 27: B & G, Patient 29: C & H, Patient 32: E & J). Flow (red arrows) and respiratory-related (yellow arrows) artefacts are diminished. Significant attenuation of the respiratory motion artefacts in two patients with highly irregular breathing pattern; green arrows (Patient 30, D & I; Patient 32: E & J). [file 12968_2021_839_MOESM2_ESM.pptx]

## Slide 1
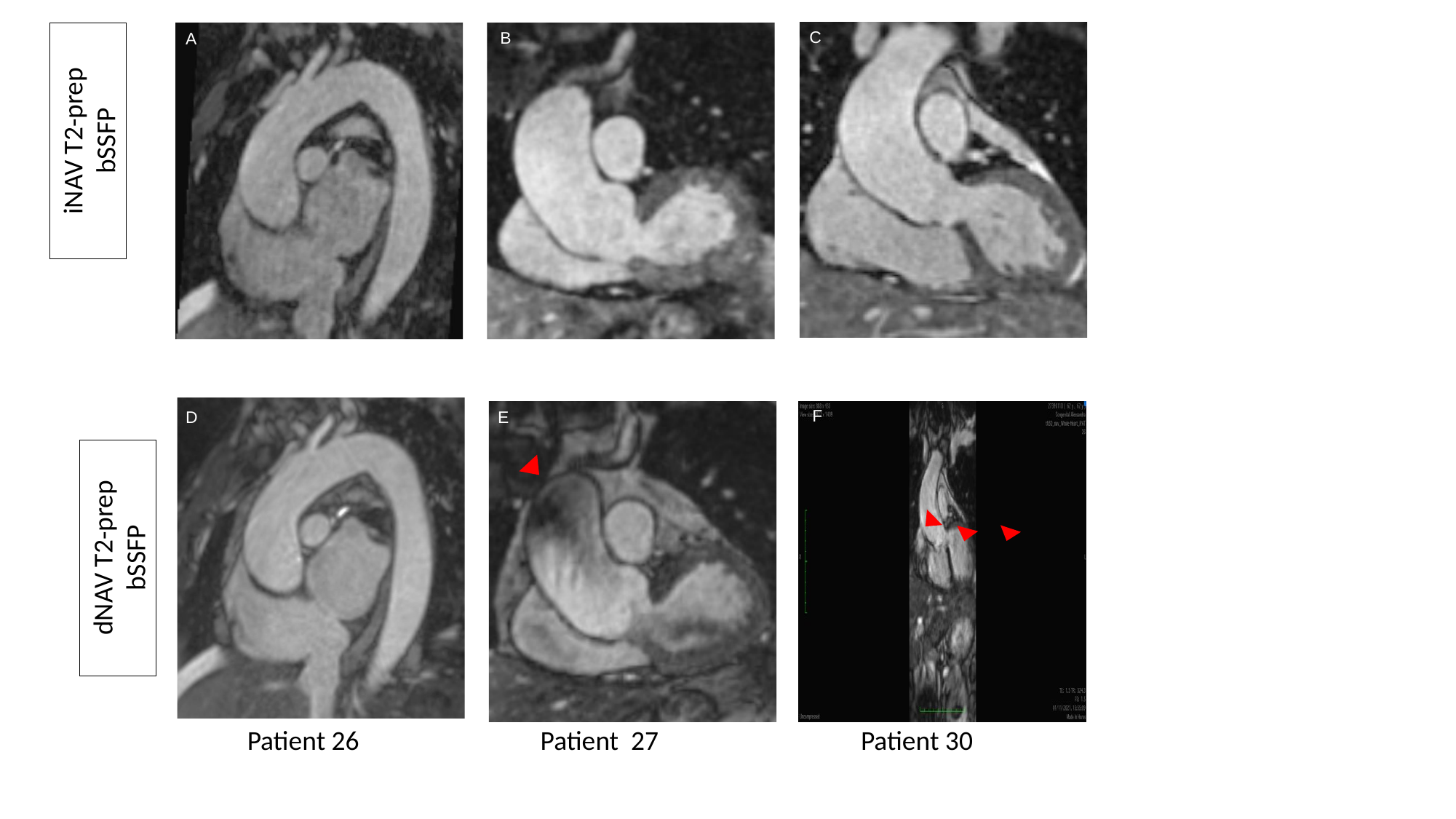

C
B
A
A
iNAV T2-prep bSSFP
F
D
E
A
dNAV T2-prep bSSFP
Patient 26
Patient 27
Patient 30

Supplement: Supplementary file 3 — Additional file 3: Figure S2. Sagittal and coronal views of the aorta for three patients with challenging acquisition. Robust aortic imaging with the iNAV T2prep-bSSFP in a patient with atrial fibrillation during the scan (Patient 26: A & D), in one patient with high BMI (Patient 27, B & E) and in one patient with highly irregular breathing pattern (Patient 30, C & F). Significant flow-related artefacts and blurring from respiratory motion (arrowheads) are alleviated. [file 12968_2021_839_MOESM3_ESM.pptx]

## Slide 1
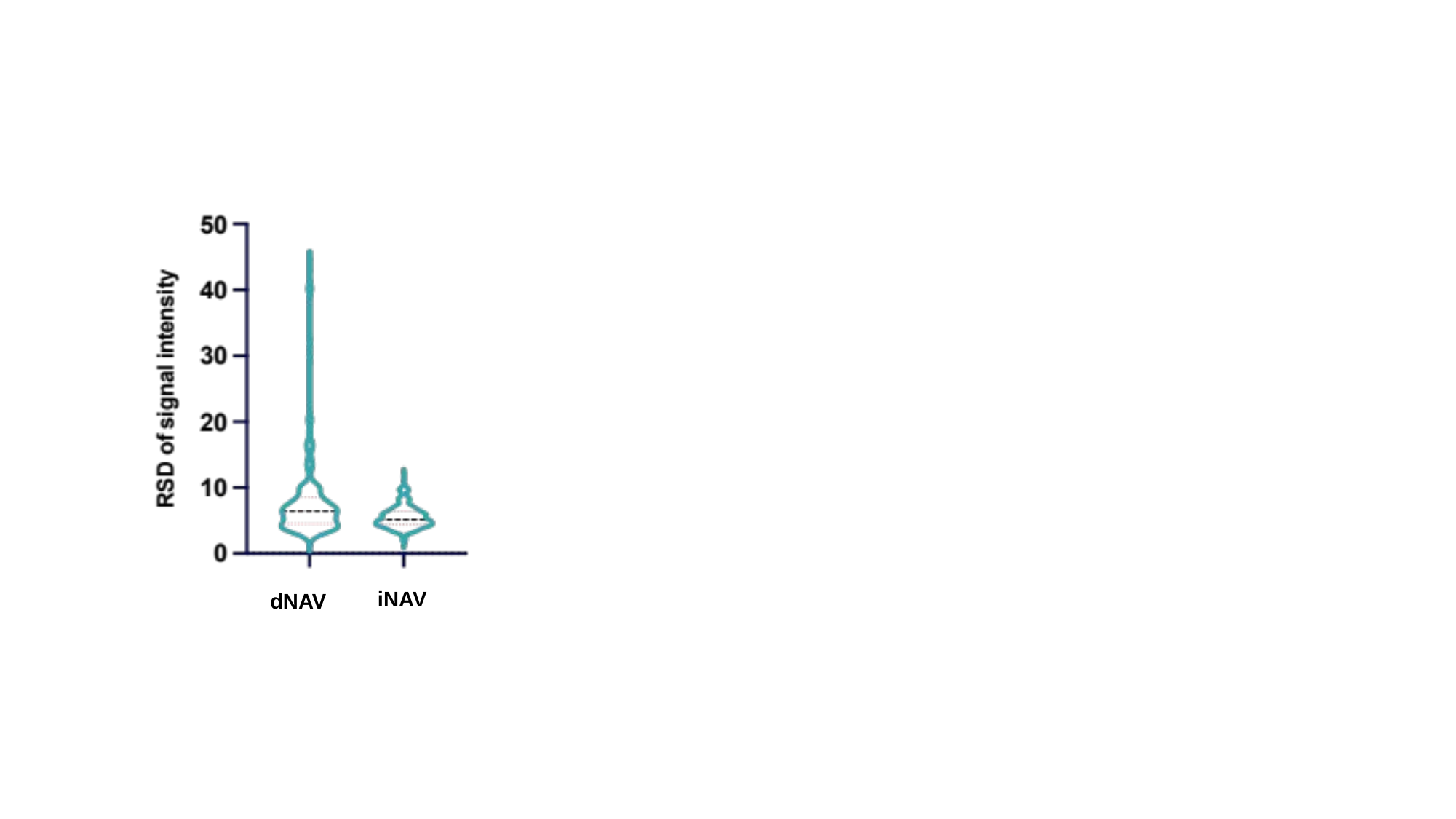

iNAV
dNAV

Supplement: Supplementary file 5 — Additional file 5: Figure S4. Relative standard deviation of signal intensity quantification in the whole thoracic aorta. Relative standard deviation of signal intensity quantification in the whole thoracic aorta for the dNAV T2-prep bSSFP and the iNAV T2-prep bSSFP. [file 12968_2021_839_MOESM5_ESM.pptx]

## Slide 1
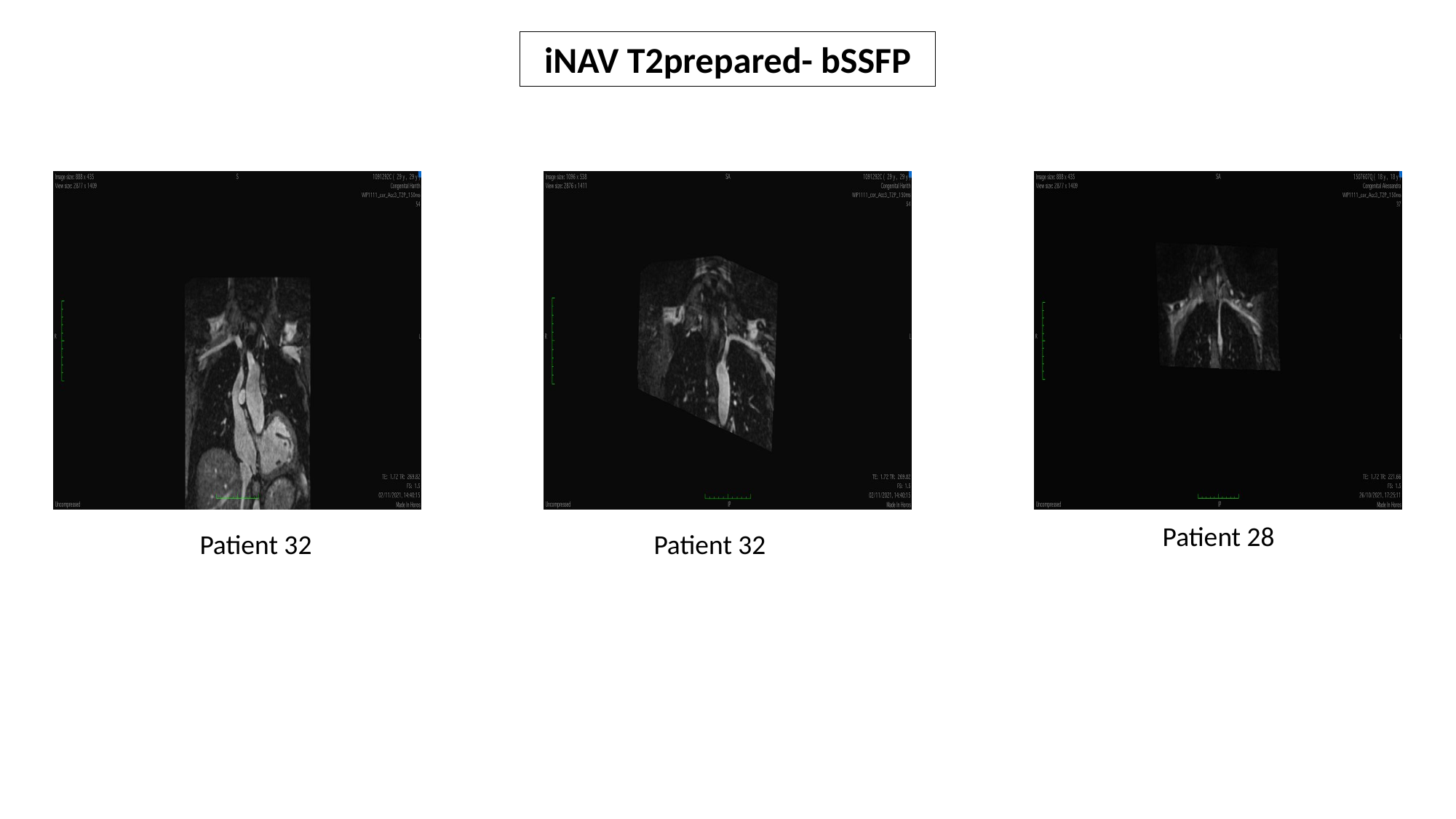

iNAV T2prepared- bSSFP
Patient 28
Patient 32
Patient 32

Supplement: Supplementary file 8 — Additional file 8: Figure S7. Coronal visualisation of the whole chest and the thoracic vasculature. Coronal orientation in planning allows visualisation of the subclavian arteries and potential aneurysms or stenosis along their entire thoracic course. [file 12968_2021_839_MOESM8_ESM.pptx]

## Slide 1
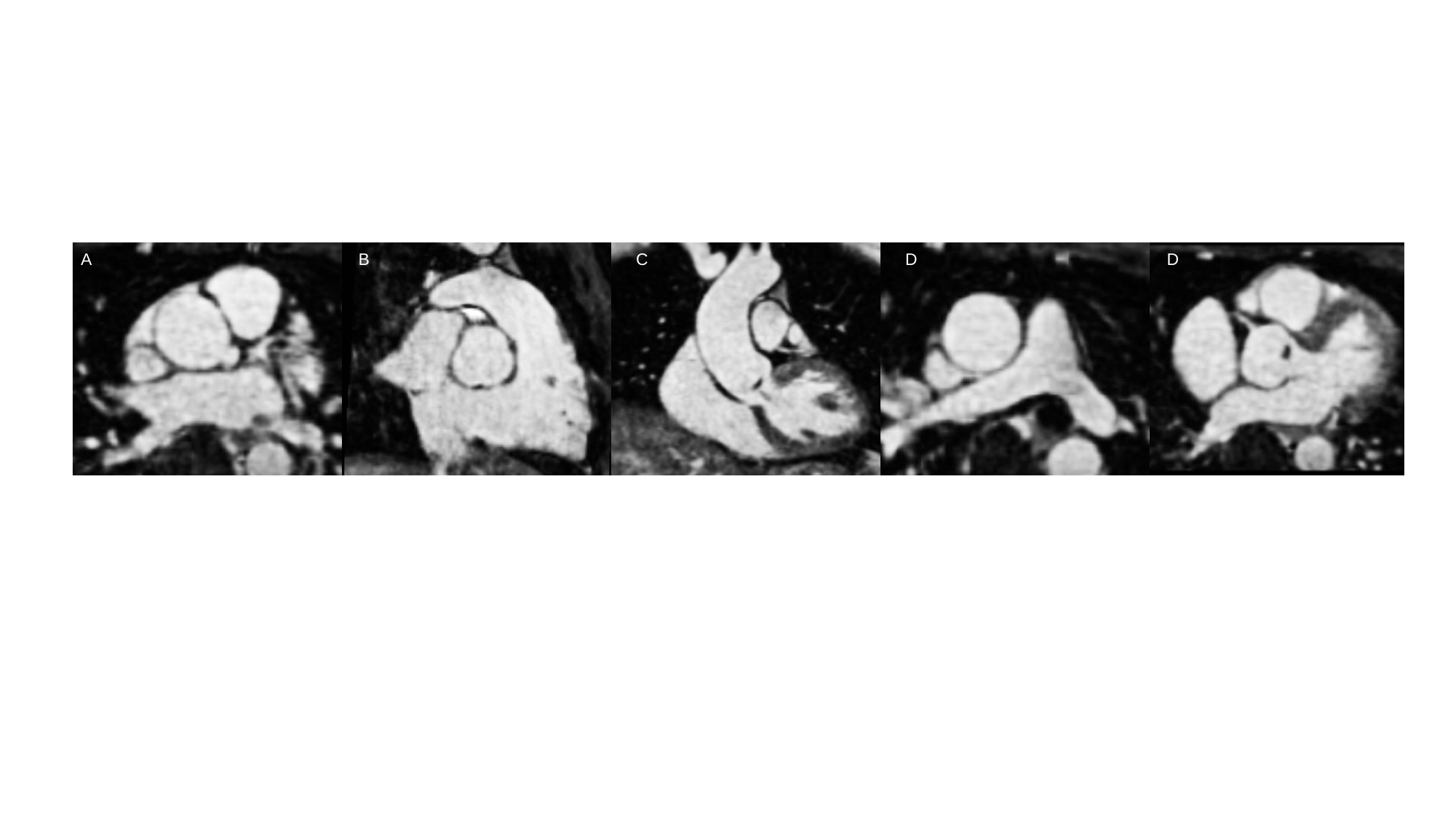

A
B
C
D
D
A

Supplement: Supplementary file 9 — Additional file 9: Figure S8. Segmental depiction of the intracardiac anatomy and the vessels connected to the heart. Segmental depiction of the pulmonary veins, the intracardiac anatomy, great arteries and coronary arteries. [file 12968_2021_839_MOESM9_ESM.pptx]
