# Supplementary material for: Efficient non-contrast enhanced 3D Cartesian cardiovascular magnetic resonance angiography of the thoracic aorta in 3 min
Source: J Cardiovasc Magn Reson. 2022 Jan 10;24:5. doi: 10.1186/s12968-021-00839-9 (PMC8744314; doi:10.1186/s12968-021-00839-9)
Supplement: Supplementary file 4 — Additional file 4: Figure S3. Example cases of different image quality visual assessment scores reported by an expert cardiologist. Example cases of different image quality visual assessment scores reported by an expert cardiologist with regards to blurring from respiratory motion (4 = severe artefact, 2 = significant artefact, 3 = mild artefact, and 4 = minimal artefact) and homogeneity of blood signal intensity (1 = non diagnostic, 2 = poor, 3 = adequate-good, 4 = excellent). [file 12968_2021_839_MOESM4_ESM.pptx]

## Slide 1
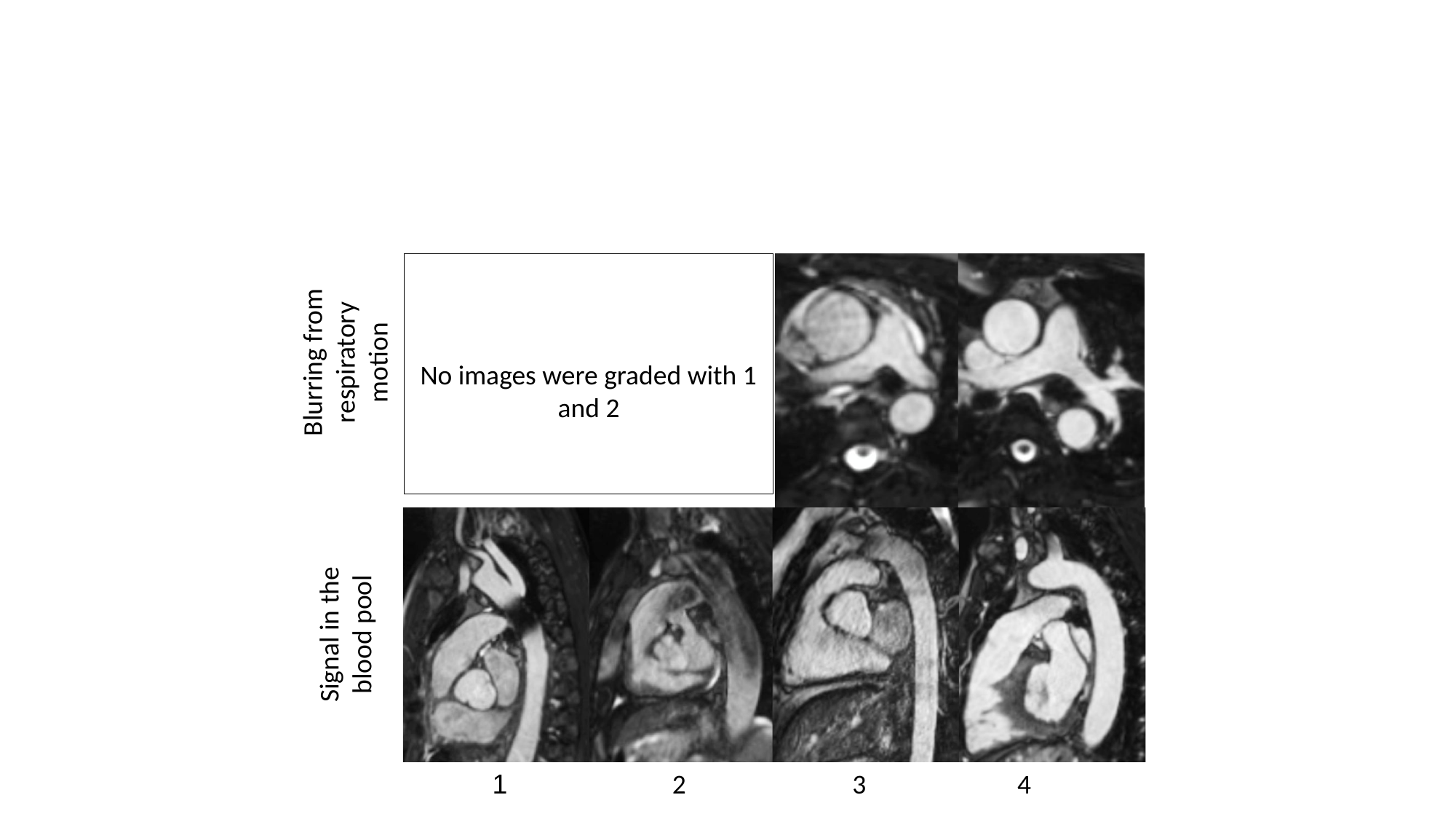

Image quality scores by the most senior expert reviewer
No images were graded with 1 and 2
Blurring from respiratory motion
Signal in the blood pool
1
2
3
4
